# Cesarean Section and Subsequent Stillbirth, Is Confounding by Indication Responsible for the Apparent Association? An Updated Cohort Analysis of a Large Perinatal Database

**DOI:** 10.1371/journal.pone.0136272

**Published:** 2015-09-02

**Authors:** Stephen Wood, Sue Ross, Reg Sauve

**Affiliations:** 1 Department of Obstetrics & Gynecology and Community Health Sciences, University of Calgary, Calgary, Alberta, Canada; 2 Canada Department of Pediatrics and Community Health Sciences, University of Calgary, Calgary, Alberta, Canada; University of Helsinki, FINLAND

## Abstract

**Background:**

Several studies and a recent meta-analysis have suggested that previous Cesarean section may increase the risk of stillbirth in a subsequent pregnancy. Given the high rates of Cesarean section in contemporary obstetric practice, this is of considerable public health importance. We sought to evaluate the potential that this association is the result of residual confounding bias.

**Methods:**

A large perinatal database (Alberta Perinatal Health Project) was searched to identify a matched set of first and second births from the years 1992–2006. Data on pregnancy outcomes, demographics and potential confounding factors were obtained.

**Results:**

The cohort was comprised of 98538 matched first and second births. Multivariate analysis did not reveal an association between previous Cesarean section and stillbirth, OR = 1.38 (0.98, 1.93). Restricting the analysis to a low risk group further attenuated the association, OR = .99 (0.62, 1.52). Analysis of the risk by indication for Cesarean section found that the risk was not increased for previous dystocia, OR = .91 (0.53, 1.55) nor for breech presentation, OR = 1.06 (0.50, 2.28) but only for other indications including non reassuring fetal status and fetal distress, OR = 1.96 (1.29, 2.98).

**Conclusions:**

The results of our cohort analysis suggest that previous Cesarean section does not cause an increased risk of stillbirth.

## Introduction

In 2003 Smith et al. reported a significantly increased risk of unexplained stillbirth in women who had delivered their first child by Cesarean section[1]. Given the importance of the outcome and the rising rates of this operation in the developed world, the results were met with a great deal of concern. Following this report, the Royal College of Obstetricians and Gynaecologists (UK) mandated that this increased risk of subsequent stillbirth be included in discussions with patients to obtain informed consent for Cesarean section. (http://www.rcog.org.uk/files/rcog-corp/Consent7_CaesareanSection09a.pdf.) Subsequent investigators, including the authors of this study, have also evaluated this issue using perinatal databases from North America, Europe and Australia with inconsistent results[2–5]. A recent systematic review included a meta-analysis of 10 observational studies documenting a pooled OR = 1.23 [95% CI 1.08, 1.40]. However, this review did not include the largest study published to date which did not find a positive association RR = 0.90 [95% CI 0.76–1.06][2]. Our research group had also reviewed these studies and had observed that important confounding factors such as maternal age, obesity, pregnancy complications and maternal medical diseases were not controlled for consistently. See S1 Appendix. Furthermore, pregnancy complications in the first birth were only controlled for in five of the studies[1,3,4,6,7]. Control for factors in the first pregnancy are particularly important as women with severe perinatal complications such as IUGR and pre-eclampsia are more likely to need a Cesarean delivery are also at risk for stillbirth in a subsequent pregnancy[8]. Our previous cohort study did not document an association between stillbirth in a second birth and Cesarean section for the first birth, OR = 1.27 [95% CI 0.92, 1.77). However, our point estimate was similar to that of the meta-analysis of O'Neill et al, OR = 1.23 [95% CI 1.08, 1.40][9]. In our first study, we did not consider the potential impact of characteristics of the first pregnancy nor the indication for Cesarean section. Therefore, we endeavoured to redress this with a re-analysis including 2 more years of data in a matched set of first and second pregnancies from the perinatal database used in our initial report[5]. Our study was approved by the Conjoint Health Research Ethics Board (ID#18311).

## Materials and Methods

Data for the study were obtained from the databases of the Alberta Perinatal Health Project (www.aphp.ca). The database contains pregnancy, delivery and pregnancy outcome data for over 350,000 births from all 81 hospitals in Alberta, Canada. Perinatal and delivery records of all perinatal deaths are reviewed by the hospital Perinatal Mortality Committee and then forwarded for further review to the provincial Reproductive Care Committee. After review, the Committee classifies all stillbirths using the Wigglesworth coding system[10] and enters the data in the electronic database. The study population was defined as women having both their first and second singleton births recorded in the perinatal database during the years 1992–2006. Information was obtained on demographic characteristics, pregnancy complications, mode of delivery, and pregnancy outcome. Smoking is recorded in the database as any during pregnancy but not the amount smoked. Weight is recorded as a over 91kg or not at first visit. Birth weight is recorded and standardized to population norms. Pregnancy induced hypertension (PIH), maternal diabetes and other medical complications were recorded in the database if diagnosed by clinicians using the standard criteria of the time. A formal definition or coding system (ICD) is not used. Information on route of delivery and indication for operative delivery is collected from the Provincial Delivery Record, a standard clinical form completed by health care providers at the time of the delivery. The primary outcome was defined, a priori, as stillbirth occurring before the onset of labour at ≥24 weeks gestation.

Analysis was planned in advance with logistic regression to control for potential confounding factors. Our first analysis was planned to control for the same variables as our previous investigation[5]: second pregnancy (maternal age(polynomial), weight>91kg, smoking during pregnancy, and maternal medical conditions(pre-existing diabetes and hypertension). These factors had been previously chosen as they are established risk factors for stillbirth. Two way interactions were also examined. Our literature review suggested an association between previous adverse perinatal outcome such as PIH, SGA, and prematurity and subsequent stillbirth[7]. Two analyses were planned. The first of these examined the possible contribution of these pregnancy complications in the first pregnancy in a model with previous cesarean section. SGA was defined as per the usual standard of <5% of birth weight and prematurity as birth <37 weeks gestation. A second analysis was also planned to restrict the analysis to only subjects who had not experienced any of these complications in their first pregnancy. A further analysis was also performed to determine if the risk of subsequent stillbirth was associated with the indication for Cesarean section. A categorical variable was created for delivery for breech presentation, arrest in labour and "other" including fetal distress and non reassuring fetal status. Breech presentation was chosen as it was felt that this group would likely have similar demographics and pregnancy complications as those with vertex presentations. Furthermore, in this group, Cesarean section would be primarily due to breech presentation so any relationship with subsequent stillbirth would be less influenced by potential confounding factors. For these latter analyses, looking at the potential residual confounding of first pregnancy factors, we planned simple crude analyses that did not include simultaneous adjustment for second pregnancy factors. Although not all readers may agree with this decision we felt that this would allow more informative assessment of the impact of including these factors than large potentially overfit models. All analyses were performed with STATA version 11. Written informed consent was not obtained from participants and all patient records were anonymized prior to analysis. The study was approved by the Conjoint Ethics Review Committee (ID#18311).

## Results

A matched set of 99650 first and second births was obtained from the Alberta Perinatal Health Database. This was a smaller data set than the population in our previous report[5], which included all second births, as it was restricted to a set where both deliveries were recorded in the database. Cases were excluded if births had gestational ages ≤23 (285 first and 257 second births), or if congenital anomalies were noted in the second pregnancy (556 cases). The final dataset included 98538 paired first and second births. The rate of previous Cesarean section was 21.1% (n = 20,765). Women who had had a previous Caesarean section were older, more likely to be obese, more likely to have pre-existing medical problems and had more complications in the first birth compared to women who previously delivered vaginally (Table 1). In the first birth 152 women had a stillbirth (1.84/1000), none had an antepartum stillbirth in their second pregnancy. There were 184 antepartum stillbirths in second births recorded in the dataset, rate = 1.9/1000. This rate is lower than expected from comparison to other Canadian and international data (typical rates 3.1–9.0/1000)[11,12]. This is likely due to the fact that the population was restricted to singleton second births <24 weeks gestation and congenital anomalies were excluded. The rates of stillbirth and cesarean section did not vary significantly over the duration of the study period (S2 Appendix) Unadjusted analysis did not suggest an increased risk of stillbirth, in second births, in women with a previous Cesarean section (OR = 1.32 [0 .93, 1.85]) p = .10.

**Table 1:** Baseline Characteristics. Matched set first and second births in Alberta 1992–2006.

	Previous Cesarean Section n = 20,765	No previous Cesarean Section n = 77,773
	n (%)	n(%)
*Maternal Characteristics*		
Age >35	3263 (15.7)	6580 (8.5)
Weight ≥91kg	2139 (10.3)	4538 (5.8)
Smoker	2720 (13.1)	12624 (16.2)
Hypertension	176 (0.9)	368 (0.5)
Diabetes	251 (1.2)	394 (0.5)
Previous SGA <3%	806 (3.9)	2382 (3.1)
Previous delivery <35 weeks gestation	748 (3.6)	1648 (2.1)
Previous PIH	1774 (8.5)	3725 (4.8)
*Outcome of Second birth*		
Cesarean Section	15178 (73.1)	4567 (5.9)
Antepartum Stillbirth	48 (0.23)	136 (0.17)
Delivery ≤35 weeks	502 (2.4)	1352 (1.7)
SGA <5%	588 (2.8)	2324 (3.0)
SGA <3%	326 (1.6)	1336 (1.7)
Neonatal Death	19 (.09)	81 (.10)

In adjusted analysis, previous Cesarean section was not associated with stillbirth when variables for risk factors in the second pregnancy (maternal age (polynomial), weight>91kg, smoking during pregnancy, and maternal medical conditions) were included (OR = 1.38 [0.98, 1.93]) p = .07. Multivariable analysis also documented an increased risk of stillbirth in the second birth with previous SGA <5% but not previous Cesarean section, pregnancy induced hypertension nor prematurity (Table 2). Interactions terms were examined but none was found to be statistically significant.

**Table 2:** Multivariable Analysis: Previous adverse pregnancy outcomes, previous Cesarean section and stillbirth in second birth.

	OR (95% CI)	P value
PIH first birth	1.28 (.74, 2.23)	.37
SGA<5% first birth	2.82 (1.85, 4.31)	< .0001
Prematurity <37weeks first birth	1.39 (.84, 2.29)	.21
Previous Cesarean Section	1.25 (.84, 1.76)	.19

Further analysis was then performed restricting the sample to a low risk group by removing subjects with previous prematurity, pregnancy induced hypertension or SGA<5%. The sample size was thereby reduced to 82645. In this low risk population, the association between previous Caesarean section and stillbirth in the second birth was null (OR = .99 [.62, 1.52]) (Table 3). The results of the restricted analysis compared to the multivariate analysis suggested that factors such as previous PIH and SGA were incompletely controlled for in the former analysis. To explore this further, we examined the data to see if there was any indication that the subjects who had Cesarean sections for SGA or PIH were different than those who delivered vaginally. In the first pregnancy, women delivered for PIH by Cesarean section were more likely to be delivered at <32 weeks gestation (5.9% vs 1.1%) and to have proteinuria (29.9% vs 22.2%). Women delivered by Cesarean section for SGA were also more likely to be delivered at <32 weeks gestation (2.8% vs 1.7%) and were far less likely to enter labour spontaneously (36.8% vs 82.3%)

**Table 3:** Crude association between previous Cesarean section and stillbirth in second birth in "low risk" subjects without previous prematurity, SGA<5% or PIH.

	Stillbirth n (n/1000)	Live Birth
Previous Cesarean Section	27 (1.62)	166683
No Previous Cesarean Section	108 (1.64)	65827

OR = .99 (.62, 1.52) p = 1.0

A planned further analysis investigated whether the specific indication for Cesarean section influences the risk of subsequent stillbirth. This analysis revealed no increased risk for subsequent stillbirth when the indication for the initial Cesarean section was breech (OR = 1.06 [.50, 2.28]), or failure to progress in labour (OR = .91 [.53, 1.55]). However, there was a significant increase in stillbirth risk when the indication for Cesarean section was in the "other" category which included fetal distress or non reassuring fetal status (OR = 1.96 [1.29, 2.98]) (Table 4). This finding was not influenced by adding factors from the second pregnancy (age, smoking, obesity and maternal medical conditions) into the model (OR = 1.99 [1.3, 3.05]).

**Table 4:** Analysis of risk of stillbirth in second birth by reason for Cesarean section in first birth. Baseline previous vaginal delivery.

Indication for Cesarean Section	OR (95%CI)	P value
Breech presentation	1.06 (.50, 2.28)	.87
Failure to progress in labour	.91 (.53, 1.55)	.73
Other (includes fetal distress, non reassuring fetal status)	1.96 (1.29, 2.98)	.0002

## Discussion

Our study sought to explore the possibility of confounding bias in the previously reported association between Cesarean section and stillbirth in a subsequent pregnancy. The findings of our updated cohort analysis causes us to question the premise that there is a causal relationship between stillbirth and previous Cesarean section. The strength of our study's conclusions are, of course, limited by those inherent in an observational design. While we are very certain that the diagnosis of stillbirths and the occurrence of cesarean section is highly accurate, data on potential confounders and the indications for cesarean section are not likely to be as precise. Still, it is not easy to envision how these inaccuracies could have produced the finding that the increased risk of subsequent stillbirth is not seen in "low risk" pregnancies and nor in women who had cesarean sections for breech babies or failure to progress in labour. We would rather expect that inaccuracies in the documentation of pregnancy complications and indications for caesarean section would make it more difficult to detect the significant associations we observed. However, it is still possible that there are factors we could not measure such as socioeconomic status or other unknown confounders that could have affected our analysis. It is also possible that our findings are not at all generalizable to other populations. In particular, we hypothesize that the more liberal the application of cesarean section, meaning the more likely low risk patients will have one, then the weaker the association between previous cesarean section and stillbirth will be.

There are 11 previous observational studies that have evaluated the risk of stillbirth with previous Cesarean section[2–7,13–17]. Of these all but one were included in the meta-analysis of O'Neill et al[9]. Five of the 10 papers included in their quantitative analysis reported a positive association between previous Cesarean section and stillbirth[3,6,7,15,17] and the other five documented null findings[4,5,13,14,16]. The largest published study to date, which included data on 11 million births, was not included in the meta-analysis and, of note, it did not find an association between Cesarean section and stillbirth [2]. It is very likely that inclusion of this study could have significantly changed the results of this meta-analysis.

Our observed rate of stillbirth, 1.9/1000 is lower than in many of the previous studies but this likely reflects differences in stillbirth definitions. Many studies that included stillbirths >20 weeks gestation and those with congenital anomalies reported rates of 4–5/1000[4,13,15]. However, our stillbirth rate was similar to that reported by Smith et al, 2.7/1000 who also defined stillbirths as ≥24 weeks gestation and excluded congenital anomalies[7]. Our restrictive definition of stillbirth limited the number of observable cases in the previous cesarean section group but we had similar numbers to previous studies[1,3,15]. Furthermore, we do not feel that low power is an explanation for our results as we were able to document a statistically significant increased risk of subsequent stillbirth in women who had a previous cesarean section for fetal distress or non-reassuring fetal status.

Inadequate control of potential confounding factors (maternal age, obesity, pregnancy complications and maternal medical diseases) was a potential source of bias in all of the previous studies, including ours. Additionally, the majority of reports provided insufficient detail about their multivariable modelling especially on the operationalization of included variables. This could be particularly important for factors such as maternal age and obesity which may not have a linear relationship with the risk of stillbirth[18–20]. The issue of the relationship between stillbirth and previous adverse pregnancy outcomes such as prematurity, intra-uterine growth restriction and pregnancy induced hypertension is particularly problematic. Surkan et al reported that previous pregnancy complications such as preterm birth, and growth restriction increase the risk of stillbirth in a subsequent pregnancy[8]. It is clear that all of these factors will also increase the risk of Cesarean section, especially as they increase in severity. Unfortunately, unless data were available to investigators on the severity of these complications, in addition to their occurrence, adequate control of potential confounding is not possible.

Therefore, a restrictive analysis removing subjects with these factors may be the best approach. In addition to our study, only one other has employed a restrictive or stratified analysis. Smith et al presented a stratified analysis to attempt to control for growth restriction in the first pregnancy.[7]. The overall analysis found that previous Caesarean section was associated with unexplained stillbirth (OR = 1.95 [95% CI 1.46, 2.60]). However, previous Cesarean section was not associated with unexplained stillbirth in women with a previous normally grown infant (OR = 1.38 [95% CI 0.93, 2.04]). Stillbirth was increased only in women with a previous small for gestational age (SGA) infant (OR = 3.34 [2.15, 5.19]).

When we further restricted our analysis to only women without previous premature births, SGA or pregnancy induced hypertension, we observed absolutely no association between stillbirth and previous Cesarean section. We also documented no increased risk of subsequent stillbirth for subjects whose indication for delivery was breech presentation or failure to progress in labour compared to those who had delivered vaginaly. The risk was only increased for those who had other indications for delivery that included fetal distress and non reassuring fetal status. There are two explanations for these findings. First, it is possible that this is evidence that only in some complicated pregnancies, does having a Cesarean section increase the risk of subsequent stillbirth. Alternatively, it is also quite possible that needing a Cesarean section is an indicator of the severity of pregnancy complications such as SGA, pregnancy induced hypertension or non reassuring fetal status. Without additional data from the perinatal databases such as Doppler ultrasound abnormalities, fetal biophysical testing or blood pressure records, the severity of these conditions cannot be adjusted for in the analysis and, therefore, may cause residual confounding. If the operation itself was increasing the risk of Cesarean section, it should increase the risk in all subjects who had the procedure. Therefore, we think that residual confounding is the most likely explanation for the association between previous Cesarean section and stillbirth but our data cannot confirm this. At the very least, our findings can be reassuring to the vast majority of women with uncomplicated pregnancies who have had a previous Cesarean section.

## Conclusions

It is our conclusion that Cesarean section is not a causal factor in stillbirth and that the positive associations previously reported are likely due to uncontrolled confounding of previous pregnancy complications. We do not feel the evidence supports the contention that the operation itself is causing stillbirth and do not think that this potential risk needs to be included in the consent process for Cesarean section. However, we do recognize that our interpretation may be incorrect and with the rapid increases in Cesarean section rates in the developed world the potential impact on stillbirth rates could be considerable. Therefore, we hope that this question will be the subject of future investigations.

## Supporting Information

S1 Appendix (DOCX)

S2 Appendix (DOCX)
