# Supplementary material for: Cesarean Section and Subsequent Stillbirth, Is Confounding by Indication Responsible for the Apparent Association? An Updated Cohort Analysis of a Large Perinatal Database
Source: PLoS One. 2015 Sep 2;10(9):e0136272. doi: 10.1371/journal.pone.0136272 (PMC4557984; doi:10.1371/journal.pone.0136272)
Supplement: S1 Appendix — (DOCX) [file pone.0136272.s001.docx]

Appendix Table 1. Previous studies examining risk of stillbirth with previous cesarean section.

| Study | Design | Population/N | Outcomes | Results | Potential Biases | Study Quality |
| --- | --- | --- | --- | --- | --- | --- |
| Smith 2003 | Retrospective Cohort.  Scottish Perinatal Database | Second singleton births 1992-98  n=120,663 | Unexplained Antepartum Stillbirth.  Total Antepartum Stillbirth. | HR= 2.33 (1.48, 3,36)* for ≥ 34 weeks  OR=1.61 (1.23, 2.11)† | No control for obesity, diabetes, or hypertension. Adjusted analysis only presented for subgroup >34 weeks gestational age. | Good |
| Taylor 2005 | Retrospective Cohort.  Australian Perinatal Database | Second singleton births 1998-2002.  n=136,101 | All Stillbirth including intrapartum. | OR= 1.10( 0.90, 1.35)* | Uncertain as to what variables were controlled for in adjusted analysis. Stillbirth only one of multiple outcomes. | Good |
| Salihu 2006 | Retrospective Cohort.  Missouri maternally linked cohort data. | Second of 2 sequential singleton births  1978-97.  n=396,441 | All Stillbirth including intrapartum. | OR= 1.1 (1.0, 1.3)* p=.40  Black Race  OR=1.4 (1.1, 1.7)* | No control for maternal medical problems. | Good |
| Bahityar 2006 | Retrospective Cohort.  US Perinatal Mortality Data. | US Singleton pregnancies >37 weeks 1995-7.  n=11,061,599 | All Stillbirth including intrapartum. | OR= .90 (0.76, 1.06)† | Limited data on potential confounders. | Good |
| Collin 2006 | Cross Sectional Survey.  Sub-Saharan Africa (22 countries) | Demographic Health Surveys 1993-2003.  n=35398 | Combined stillbirth, miscarriage and therapeutic abortion. | OR=1.3 (0.8, 1.9)* | Response rate unclear. Outcomes determined by self report. | Poor |
| Smith 2007 | Retrospective Cohort.  Scottish Perinatal Database | Second singleton births 1992-2001.  n=133000 | Total Antepartum Stillbirth  Unexplained Stillbirth | OR=1.96 (1.53, 2.49)†  HR=1.75 (1.30, 2.37)* | No control for obesity, diabetes and hypertension. Details on modelling not provided. | Good |
| Richter 2007 | Retrospective Cohort.  Berlin Perinatal Database. | Second births 1993-99.  n= 62698 | Antepartum Stillbirth. | OR=1.52 (1.04, 2.20)* | Crude analysis not statistically significant. No details on modelling for adjusted analysis. | Good |
| Gray  2007 | Retrospective Cohort.  Oxfordshire (UK) Perinatal Database. | All singleton births 1968-89.  n=81784 | Total Stillbirth.  Unexplained Stillbirth. | HR=1.54 (1.04, 2.29)†  HR=1.19 (0.62, 2.13)† | Full adjusted analysis only possible for 49% of subjects due to missing data. | Good |
| Kennare 2007 | Retrospective Cohort  South Australian Perinatal Database | All second singleton births 1998-2003.  n=36038 | Total Stillbirth  Unexplained Stillbirth | OR=1.56 (1.04,2.32)*  OR=2.34(1.26, 4.37)* | Crude analysis non significant. Very limited number of covariates in adjusted analysis. | Good |
| Wood 2008 | Retrospective Cohort  Alberta (Canadian) Perinatal Database | All second singleton births 1991-2004  n=157929. | Total Stillbirth  Antepartum Stillbirth  Unexplained Stillbirth | OR=1.17(0.96, 1.42) †  OR=1.09(0.86, 1.39) †  OR=1.27(0.92, 1.77) * | No data on socioeconomic status or past pregnancy complications. Incomplete control of obesity. | Good |
| Franz 2009 | Retrospective Cohort  Bavarian Birth Records. | All second singleton births.  n=629815 | Total Stillbirth | OR=1.0 (0.86, 1.16) † | Misclassification of previous caesarean section in 25% of subjects. | Poor |
| Ohanna 2011 | Retrospecitve Cohort  Single centre Israel. | All births. n=228293 | Antepartum Stillbirth | OR=1.4(1.2, 1.6)†  OR=0.8 (0.7, 0.9) * | Analysis included all births including first births. | Good |

*Adjusted estimates †Unadjusted estimates
