# Supplementary material for: Cesarean Section and Subsequent Stillbirth, Is Confounding by Indication Responsible for the Apparent Association? An Updated Cohort Analysis of a Large Perinatal Database
Source: PLoS One. 2015 Sep 2;10(9):e0136272. doi: 10.1371/journal.pone.0136272 (PMC4557984; doi:10.1371/journal.pone.0136272)
Supplement: S2 Appendix — (DOCX) [file pone.0136272.s002.docx]

Appendix Table 2. Cesarean Section rates and stillbirth rates by year.

| Year | Cesarean Section  Rate % in first births | Stillbirth Rate (n/1000)  In second births |
| --- | --- | --- |
| 1992 | 16.19 | 0 (n=1) |
| 1993 | 16.32 | 1.5 |
| 1994 | 17.77 | 2.8 |
| 1995 | 17.20 | 2.3 |
| 1996 | 18.36 | 1.2 |
| 1997 | 18.00 | 1.6 |
| 1998 | 18.25 | 1.6 |
| 1999 | 20.23 | 2.6 |
| 2000 | 21.61 | 1.1 |
| 2001 | 22.99 | 1.7 |
| 2002 | 24.28 | 1.1 |
| 2003 | 24.72 | 1.6 |
| 2004 | 23.55 | 1.3 |
| 2005 | 23.52 | 2.8 |
| 2006 | 40% (n=2) | .6 |

Note: Cesarean Section rate is for first births and stillbirths are for second births. Cesarean section rate data for 2006 are based on small number (n=5) as subjects who had a first birth in the last year data was available would need to have a second birth that year to be included in the study. Stillbirth data for 1992 is likewise limited (n=1)
